# Numerically Precise Benchmark of Many-Body Self-Energies on Spherical Atoms

**DOI:** 10.1021/acs.jctc.2c00048

**Published:** 2022-05-13

**Authors:** S. Vacondio, D. Varsano, A. Ruini, A. Ferretti

**Affiliations:** † Dipartimento di Scienze Fisiche, Informatiche e Matematiche, Università di Modena e Reggio Emilia, Via G. Campi 213/a, Modena 41121, Italy; ‡ Centro S3, CNR−Istituto Nanoscienze, 41125 Modena, Italy

## Abstract

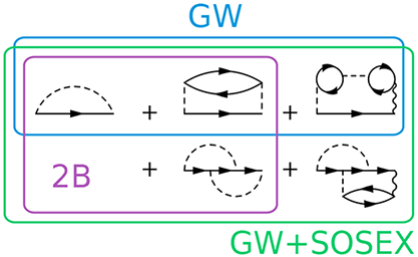

We investigate the performance of beyond-GW approaches in many-body perturbation theory by addressing atoms described within the spherical approximation via a dedicated numerical treatment based on B-splines and spherical harmonics. We consider the GW, second Born (2B), and GW + second order screened exchange (GW+SOSEX) self-energies and use them to obtain ionization potentials from the quasi-particle equation (QPE) solved perturbatively on top of independent-particle calculations. We also solve the linearized Sham–Schlüter equation (LSSE) and compare the resulting xc potentials against exact data. We find that the LSSE provides consistent starting points for the QPE but does not present any practical advantage in the present context. Still, the features of the xc potentials obtained with it shed light on possible strategies for the inclusion of beyond-GW diagrams in the many-body self-energy. Our findings show that solving the QPE with the GW+SOSEX self-energy on top of a PBE or PBE0 solution is a viable scheme to go beyond GW in finite systems, even in the atomic limit. However, GW shows a comparable performance if one agrees to use a hybrid starting point. We also obtain promising results with the 2B self-energy on top of Hartree–Fock, suggesting that the full time-dependent Hartree–Fock vertex may be another viable beyond-GW scheme for finite systems.

## 1 Introduction

The GW approximation^1−3^ of many-body perturbation theory (MBPT) has been the state-of-the-art method for the prediction of excitation spectra for the last 40 years.^4−7^ It has been applied to the band structure calculation of a variety of condensed matter systems, with the band gap being the property best predicted. Over the last 15 years, it has also found a successful application in molecules.^8−14^ GW, however, still fails at predicting a number of properties and phenomena, such as occupied bandwidths,^15−23^ satellites,^24^ strong correlations,^25^ and orbital energy ordering and spacing.^26−28^ It being framed within Hedin’s iterative scheme, a self-consistent approach to GW should be implemented in principle. In practice, this is often detrimental for the prediction of band structures and energy levels,^29,30^ so different flavors of self-consistency are pursued instead,^21,31,32^ including no self-consistency at all, that is, single shot G_0_W_0_ calculations. This gives rise to a starting point dependence of GW, which is also numbered among its drawbacks.

In Hedin’s iterative scheme, GW corresponds to the neglect of vertex corrections. In order to fix the shortcomings of GW, there has been a recent resurgence of interest in the field of vertex corrections. While it has been suggested that vertex corrections may have little effect on the band gap prediction,^33^ they have been found to improve other observable quantities such as ionization potentials of extended systems^34,35^ and molecules.^27,36,37^ The ambiguity concerning self-consistency has also been addressed: By now it has been accepted that vertex corrections and self-consistency effects tend, at least partially, to cancel out,^23,38,39^ justifying non-self-consistent GW calculations on the one hand and suggesting that full self-consistency should be pursued only in the presence of vertex corrections on the other. There is no unambiguous way to go beyond GW. Following Hedin’s scheme, the most natural approach would be to simply take the first self-energy diagram of higher order than GW in the screened interaction.^33,34,38^ Alternative approaches include those targeting positive-definiteness of the spectral function^40−42^ or compliance with the Ward identity.^20,35^ For computational simplicity, local 2-point vertices have also been proposed.^35,43−45^

In the present work, we benchmark low-order expansions of self-energies obtained by employing nontrivial vertex functions (see Appendix A). In particular, besides the GW approximation, we consider the second Born (2B) self-energy^46^ and the GW plus second order screened exchange (GW+SOSEX) self-energy introduced in refs (27 and 47). We use spherical atoms as our test bed, reducing the numerical problem to one spatial dimension and treating it with a dedicated all-electron implementation based on a B-spline basis and spherical harmonics. The simplicity of the problem allows us to avoid the use of a number of approximate techniques commonly adopted in general-purpose codes, including pseudopotentials, frequency representations,^48,49^ and extrapolations to the complete basis set limit. The numerical error stemming from these approximations may hinder the assessment of method accuracy and makes the comparison of results from different codes difficult. Moreover, assuming a negligible contribution from relativistic effects, the numerical precision we achieve allows for a direct comparison with experiments. To our knowledge, in the literature there are few MBPT studies focused on atoms,^8,9,50−53^ while some general MBPT benchmarks include atomic systems as a part of larger test sets.^54,55^

Another important aspect to be assessed when using self-energy-based methods is the study of the optimal starting point and possibly even of the effect of self-consistency. If on the one hand 2B is naturally framed within the time-dependent Hartree–Fock (TDHF) approach^8,36,37^ and is therefore fit for a HF reference, on the other hand, GW and GW+SOSEX have shown more ambiguity. In molecules, GW seems to work best on average with a hybrid DFT starting point.^11,14,26^ This can be explained by the fact that HF constitutes a decent guess for orbitals and eigenvalues to start with but offers a poor description of screening in self-energies containing a random phase approximation (RPA) screened interaction such as GW. As already witnessed in solid state systems, the RPA screening tends to perform much better when built on the KS system,^56^ which however constitutes an especially bad guess for energy levels in molecules, where local and semilocal functionals miss the 1/*r* decay of the xc potential.^57,58^ Therefore, hybrid functionals such as PBE0 should in principle bring in the best of both worlds, but this is not always true: In fact, the HF reference and different flavors of self-consistent schemes have also been considered in specific cases and have proven better than a hybrid reference.^52,54,59−61^ GW+SOSEX has so far shown less ambiguity, performing much better with PBE and PBE0^27,62^ rather than with HF;^28^ self-consistent schemes have only been marginally addressed with this self-energy.^63^

In this work, other than considering the usual HF, PBE, and PBE0 references in perturbative (or “one-shot”) many-body calculations, we also address self-consistency in a less traditional way by employing the self-consistent solution of the linearized Sham–Schlüter equation (LSSE)^64^ as a starting point. As shown by our results, in atoms this constitutes a privileged starting point whose highest occupied molecular orbital (HOMO) is left mostly unchanged by a single iteration of the Dyson equation, provided that the self-energy used in the Dyson equation is the same as that used in the LSSE.^65,66^ This is a strong hint that, as is expected,^67^ the density of the starting point is largely preserved, given the relation that HOMO energy levels have with the decay of the density.^68,69^ Band gaps similar to those of self-consistent GW have been found in condensed matter by adopting this procedure,^70^ and a good agreement with experiment has also been found for selected systems.^71^

Given the availability of very accurate xc potentials for selected atoms,^58,72^ we also propose the solution of the LSSE as a further benchmark for self-energies, as is also done with total energy functionals.^73^ In this respect, the LSSE can be considered an alternative, if not an approximation, to the self-consistent Dyson equation of MBPT. In fact, both approaches are based on the stationarity of the Klein energy functional, the latter with an unconstrained Green’s function (GF) and the former under the restriction of the GF being noninteracting and subject to a local potential.^67^ The accuracy of the LSSE as an approximation to the self-consistent Dyson equation has not been thoroughly investigated yet. Solving the LSSE with the HF self-energy has been found to yield good approximations to the HF HOMOs, and the upper valence thus obtained is also in closer agreement with HF than one would get with local and semilocal KS-DFT functionals.^74,75^ In this case the static, spatially nonlocal Fock exchange self-energy is approximated with a static, spatially local xc potential. In general, however, the xc potential is required to approximate dynamical, spatially nonlocal self-energies obtained in the self-consistent solution of the Dyson equation, which may be too demanding. As of now, an argument involving the adiabatic connection between the self-consistent Dyson equation and the self-consistent KS equations has been proposed in order to justify the LSSE solution as a starting point for many-body calculations (see Appendix B of ref (65)): solving the Dyson quasi-particle equation on top of it (without renormalization factor) provides a first-order approximation to the self-consistent quasi-particle energies.

In general, our results suggest that despite the consistency that the LSSE starting points display for many-body calculations these are not a good approximation to the self-consistent Dyson equation. The numerical effort required for the solution of the LSSE is not even justified by an increased accuracy with respect to traditional perturbative schemes starting from (generalized) KS-DFT solutions. In fact, when the GW and GW+SOSEX self-energies are used, we find it beneficial to use such perturbative schemes, which are known to generally render the RPA screened interaction *W* reasonably accurate as discussed earlier in this section. Confirming the common knowledge on finite systems,^11,14,26^ we find that even in atoms the GW self-energy needs a fraction of nonlocal exchange in the KS-DFT starting point to achieve good accuracy, which we obtain with the PBE0 functional. Instead, the GW+SOSEX self-energy displays no such need, also showing a reduced starting point dependence in going from PBE to PBE0.^27^ The 2B self-energy is also capable of yielding good, if not excellent, accuracy in many atoms at a reduced computational cost, provided that the HF starting point is used. This draws our interest to the full TDHF vertex.

This article is organized as follows: In section 2, we present the two ways in which we deal with self-energies in this work: by plugging them either in the many-body QPE or in the LSSE. Then we introduce the self-energies under investigation and briefly detail how we perform frequency integrations. We present and discuss the results in section 3, starting from the exposition of the main numerical aspects of our treatment (section 3.1). Then, given the specificity of the approach, we devote section 3.2 to elucidating the features of the xc potentials obtained from the self-consistent solution of the LSSE. Next, in section 3.3, we assess the performance of the selected self-energies by looking at ionization potentials (IPs) as obtained from the negative of the HOMO energies. Finally, we draw our conclusions in section 4 and elaborate on the possible forthcoming work.

## 2 Theoretical Framework

### 2.1 The Green’s Function and the Dyson Equation

The mathematical key object of MBPT is the one-particle Green’s function (GF), from which the charged excitation spectrum of a system can be extracted. Starting from the Hartree GF, *G*_H_, the GF of the system of interacting electrons *G* can be obtained via the Dyson equation,^76^
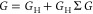
1involving the self-energy operator Σ. The self-energy can be cast as a functional of the interacting GF, that is, Σ = Σ[*G*], in order to write approximations to Σ as sums of a few physically meaningful Feynman diagrams.

Having Σ = Σ[*G*] implies that the Dyson equation should in principle be solved self-consistently. In practical implementations, the calculation of *G* is often perturbative (also termed “one-shot”), consisting of a single iteration of the Dyson equation upon an independent-particle solution of the electronic problem. Here we follow this path starting from either a HF or a KS-DFT GF, whereas in the next section we will tackle self-consistency by letting Σ = Σ[*G*] in the Sham–Schlüter equation (although we will quickly approximate *G* with *G*^KS^). Therefore, the Dyson equation reads either
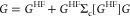
2when starting from a HF solution or
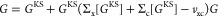
3when starting from a KS-DFT solution. In the latter case, the xc potential, *v*_xc_, is removed and the nonlocal Fock exchange, Σ_x_, is added; in the former case, the Fock exchange is already present in *G*^HF^, and only the correlation self-energy, Σ_c_ = Σ – Σ_x_, is needed in going from the noninteracting *G*^HF^ to the interacting *G*. From the GF *G* thus obtained, the spectral function
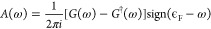
4can be computed, and its peaks can be identified with the charged excitations of the system. Here ϵ_F_ is the Fermi energy. The operator *A* is meant to reproduce the spectra from angle-resolved photoemission experiments, its trace representing the density of states. In the absence of strong correlation, the main peaks of the spectral function are found in correspondence of the so-called quasi-particle (QP) energies, ϵ^QP^. For numerical convenience, a diagonal approximation of the GF, self-energy, and spectral function operators is often adopted, yielding the quasi-particle equations (QPEs),
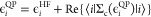
5and
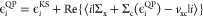
6for the computation of QP energies with the HF and the KS-DFT starting points, respectively. The *i* index runs over the independent-particle states of the system, to which QP states correspond one by one. We refer to the difference between the QP energy and the independent-particle energy as QP shift.

### 2.2 The xc Potential and the Sham–Schlüter Equation

Both MBPT and KS-DFT are capable of yielding the exact ground-state electron density of a system, provided that the exact self-energy Σ and the exact xc potential *v*_xc_ are used. Applying to both sides of eq 3 (now with Σ = Σ[*G*]) a linear density operator , which maps *G* to the ground-state density of the system, yields
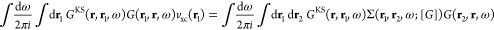
7which is known as the Sham–Schlüter equation (SSE).^64^ The density condition employed for deriving it implies that *G*^KS^ produces the same density as *G*. The equation is in the form *Ax* = *b*, and can be solved as a linear system in the unknown *x*, which is the xc potential, *v*_xc_(**r**). Since the unknown itself is needed to compute *G*^KS^, which enters the kernel *A* and the inhomogeneous term *b*, the problem must, in general, be solved iteratively. Most often, it is linearized by performing the substitution *G* → *G*^KS^, which amounts to retaining the lowest order in *G*^KS^ in eq 3. By doing so one obtains the linearized Sham–Schlüter equation (LSSE):
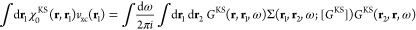
8where χ_0_^KS^ is the KS independent-particle static polarizability. We emphasize that the dependence Σ = Σ[*G*] in the full SSE implies that the full Dyson equation for *G*, eq 1, is obeyed self-consistently, while this is not the case for the LSSE, where Σ = Σ[*G*^KS^] and there is no reference to *G* any longer. This implies that the density condition used to derive the SSE no longer holds exactly, and it has to be verified how close the densities produced by the fully self-consistent *G* and by the LSSE-self-consistent *G*^KS^ are. The KS scheme differs significantly from the MBPT scheme, in that it does not account for frequency dependence and is affected by the KS gap problem.^77−79^ Nonetheless, one iteration of the Dyson equation is expected to preserve the density of *G*^KS^ as obtained from the self-consistent LSSE.^65−67^

Alternatively, the LSSE can also be derived via an optimized effective potential (OEP) strategy starting from total energy functionals.^67,80,81^ In the framework of MBPT, it is possible to build total energy functionals that depend directly on the GF.^82,83^ In this case, the LSSE can be obtained^67,84^ by minimizing the Klein functional with the constraint that the GF be given by a static and spatially local potential. The Klein functional reads^83,85−88^
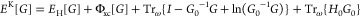
9and its (unconstrained) optimization implies that the Dyson equation is obeyed self-consistently at stationarity. In this expression, Tr_ω_{} stands for , *E*_H_ is the Hartree energy and *G*_0_ is the free-particle GF corresponding to *H*_0_ = *T* + *v*_ext_. The Φ_xc_[*G*] functional, which carries electron interaction contributions beyond Hartree, is also related to the self-energy by
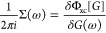
10Constraining the Klein functional to GF *G*_s_ obtained from a static and spatially local potential *v*_s_(**r**) yields
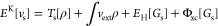
11where *T*_s_ is the kinetic energy of noninteracting particles (subject to a local potential) of density ρ. Imposing the variational condition  yields
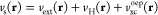
12where *v*_xc_^oep^(**r**) is obtained as the solution of the LSSE, eq 8. Importantly, as in the unconstrained optimization, the self-energy in the LSSE/OEP case is obtained from the Φ functional via eq 10. Nevertheless, Σ is no longer a functional of *G* but rather of *G*^KS^ (see eqs 7 and 8).

### 2.3 Self-Energies

**Figure 1:**
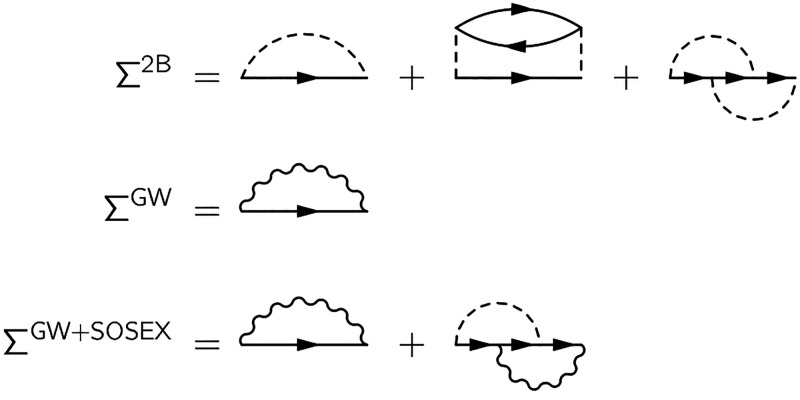
Self-energy diagrams benchmarked in this work. Solid lines represent the fermion propagators, dashed lines the bare Coulomb interaction, and wiggly lines the dressed Coulomb interaction.

In the present work, we focus on the second Born (2B), GW, and GW plus second-order screened exchange (GW+SOSEX) self-energies. As seen in the first line of Figure 1, besides the Fock exchange, the 2B self-energy includes a second order direct (2Bd) and a second order exchange (2Bx) diagram. These are all possible second order irreducible diagrams. As long as occupied states are concerned, they balance each other with respect to the self-screening error, meaning that solving the Dyson equation with this self-energy in a one-electron system yields a vanishing QP shift of the occupied level. On the other hand, in 2B, screening is accounted for through the use of one bubble in the direct diagram, making this self-energy unsuitable for extended systems (especially metals) and possibly large polarizable molecules.

Screening is better accounted for within the GW approximation (second line of Figure 1), where it is introduced at the random phase approximation (RPA) level. This comes with the price of having only a direct diagram in the self-energy, giving rise to a self-screening error.^88−90^ Ameliorating the situation with the inclusion of the bare 2Bx diagram, balancing the 2Bd diagram naturally contained in GW, is unsuitable in extended systems and has proven unsuccessful even in molecules.^26^ A partial inclusion of RPA screening at second order via the SOSEX diagram (third line of Figure 1) has led to more promising results,^27,62,63^ and the diagram itself can be justified by symmetry arguments^27^ originally made for RPA total energies.^91,92^ These arguments aim at restoring the antisymmetry of the two-particle GF upon odd permutation of its space–time arguments (also referred to as crossing symmetry), as the fermionic nature of electrons requires. A justification of the SOSEX diagram within the T-matrix formalism is also possible.^47^ It must be noted that even with the SOSEX diagram full cancellation of the self-screening error occurs only up to second order in the bare Coulomb interaction, that is, at the 2B level of theory. It is also unknown whether the partial screening in this diagram can actually make it suitable for metals.

We now give the analytical expressions for the self-energies, in a real-space/frequency representation. The 2B self-energy reads
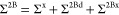
13with Σ^x^ being the Fock self-energy,
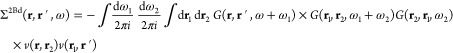
14and
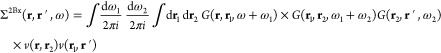
15*v* is the Coulomb interaction. The GW self-energy reads:
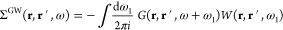
16Finally, the SOSEX contribution to the GW+SOSEX self-energy reads
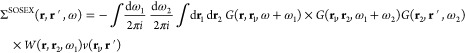
17with the GW+SOSEX self-energy obviously given by Σ^GW+SOSEX^ = Σ^GW^ + Σ^SOSEX^. All the screened interactions *W* are to be intended in the RPA, that is, the Dyson equation,
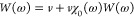
18is obeyed with χ_0_ being the independent-particle irreducible polarizability, that is,
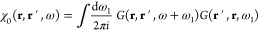
19

**Figure 2:**
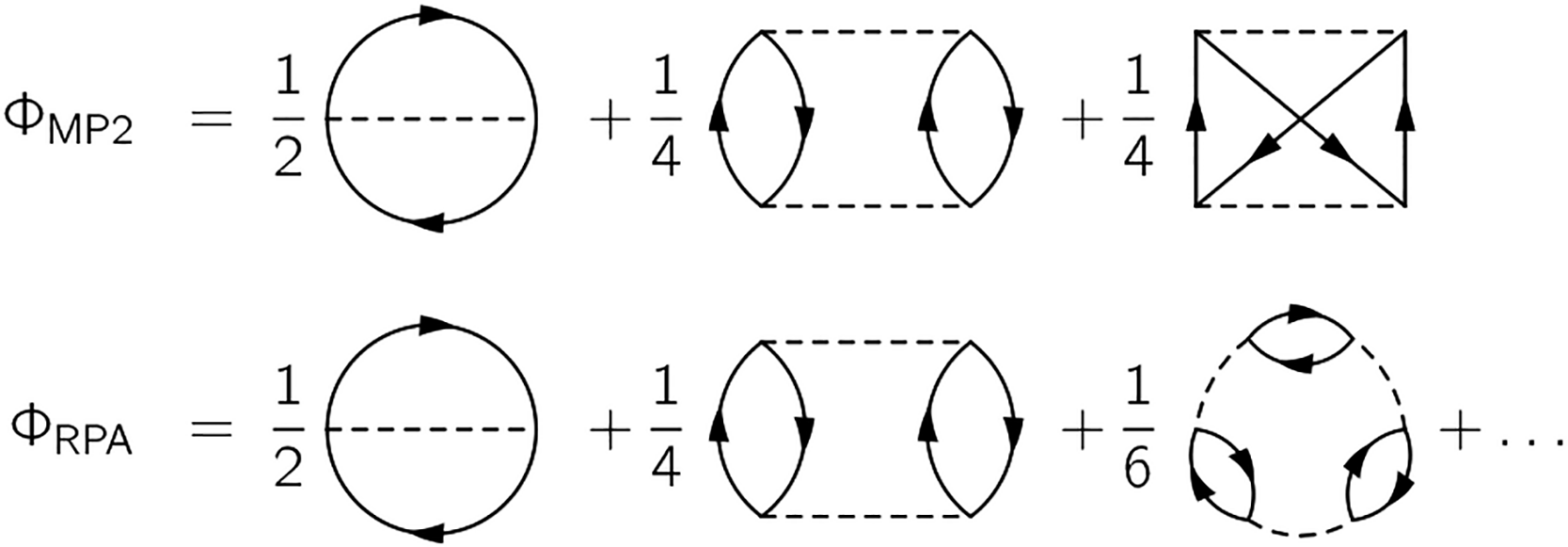
MP2 and RPA Φ functionals, from which the 2B and GW self-energies, respectively, are obtained upon differentiation with respect to the GF.

We conclude this section by linking the self-energies to their respective total energy functionals, when available. Approximate KS-DFT total energies can be obtained via the constrained Klein functional by making approximations on the Φ functional (see eq 11). We remark that even with the exact Φ functional, the exact energy cannot be obtained as long as KS GFs are fed to the Klein functional (therefore making it the constrained Klein functional). An approximation on the Φ functional in turn entails an approximation on the self-energy (see eq 10). In particular, the Φ functional yielding the HF self-energy upon differentiation (first diagram of either line in Figure 2) leads to the exact-exchange (EXX) energy upon insertion in eq 11. Following this reasoning, the 2B self-energy corresponds to the MP2 energy (first line in Figure 2), and the GW self-energy corresponds to the RPA energy (second line in Figure 2). On the other hand, the GW+SOSEX self-energy (at least in the current formulation with *W* treated at the RPA level) is not Φ-derivable and is therefore not rigorously suitable for the variational argument presented in section 2.2. The total energy expressions termed RPA+SOSEX introduced in the frameworks of coupled cluster theory^91^ and of the adiabatic connection formula^92^ are not formally linked to the GW+SOSEX self-energy. We nonetheless apply the LSSE treatment to the GW+SOSEX self-energy for benchmarking purposes.

For coherence with this picture and the literature, we adopt the following nomenclature: we will refer to the potential obtained from the HF self-energy through the solution of the LSSE as the EXX potential, the potential obtained from the 2B self-energy will be the MP2 potential, and the one obtained from the GW self-energy will be the RPA potential. On the other hand, the GW+SOSEX self-energy will simply correspond to the GW+SOSEX potential.

### 2.4 Frequency Integrations

Implementing the solution of the Dyson equation in a one-shot fashion greatly simplifies the numerical problem at hand. In this case, all the GFs appearing in eqs 13–19 can be taken to be noninteracting and cast in a spectral representation by using the orbitals ψ_α_ and eigenvalues ϵ_α_ of the underlying independent particle Hamiltonian, according to
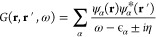
20where η is a positive, vanishingly small real value ensuring the time-ordering expression of the GF; we use Greek letters to indicate suitable multi-indices. Frequency integrals can then be easily performed by means of the residue theorem, when the explicit knowledge of the poles of *W* is not required. In fact, while it is in principle possible to cast *W* as a sum over poles, for example, by solving an eigenvalue problem,^93^ here we solve eq 18 via a matrix inversion at given frequencies in the complex plane, so we do not have access to the explicit position of the poles of *W*. Before proceeding, we introduce the following notation for partially and fully saturated Coulomb integrals, respectively:
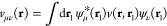
21
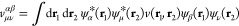
A similar notation is also used for *W*(ω).

Next, we consider frequency integrations for each self-energy and see how the frequency dependence of *W* is dealt with, when present. In the case of the 2B self-energy, there is no *W* and the solution of the frequency integrals using the residue theorem simply yields
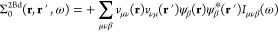
22
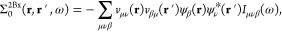
23with
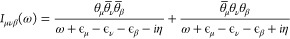
24We have also set θ_μ_ = θ(ϵ_μ_ – ϵ_F_) and θ̅_μ_ = 1 – θ_μ_ = θ(ϵ_F_ – ϵ_μ_). We place 0 in the subscripts of self-energies to indicate that they are computed with a one-shot procedure.

In the case of GW, the frequency integration involving *W* can be dealt with using a contour deformation technique.^94,95^ The same can be done with the SOSEX diagram after carrying out the integral in ω_2_ of eq 17; by doing so using the residue theorem, one gets
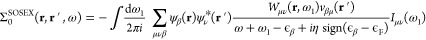
25with
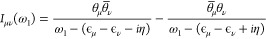
26The fact that *I*_μν_ has the same pole structure as *W* allows for the application of the contour deformation technique.

When Σ enters the LSSE (eq 8), the frequency integral in the RHS, that is, the inhomogeneous term, can be again performed using the residue theorem, and the integrals involving *W* that were treated using the contour deformation simply reduce to integrals over the imaginary axis.^96,97^ As an example, we consider the frequency integral in the correlation contribution to the RPA inhomogeneous term, obtained when the GW self-energy is used:
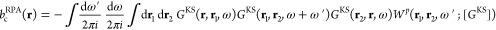
27with *W*^p^ = *W* – *v*_c_, and we stress that is obtained from *G*^KS^ in this last expression. Expressing each GF in spectral form according to eq 20 (also dropping the KS label for ease of notation), and integrating with respect to ω the three GF of eq 27, one obtains
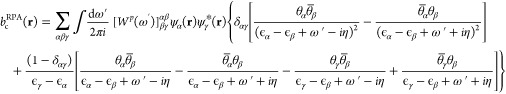
28The fact that this expression has the same pole structure as *W* allows once again for integration with respect to ω′ in eq 27 via the contour deformation technique, which in this case trivially reduces to an integration over the imaginary frequency axis. The result in eq 28 can also be obtained for the MP2 and SOSEX inhomogeneous terms. While for the latter a continuation to the imaginary axis is again applied to compute the leftover frequency integral, a fully analytical expression can be worked out for the former using the residue theorem.

## 3 Results and Discussion

### 3.1 Numerical Details

The numerical problem for spherical atoms can be reduced to one dimension, namely, the radial one. The leftover angular degrees of freedom can be treated analytically by means of the spherical harmonics machinery. In other words, matrices are made block diagonal in the spherical harmonics representation. To give a flavor of how the angular problem is treated, we mention that the coupling of spherical harmonics in direct self-energy diagrams such as 2Bd and GW yields the Wigner 3j symbols, whereas in exchange diagrams such as 2Bx and SOSEX, the Wigner 6j symbols appear. The basics of this machinery can be found in ref (98).

The numerical radial problem is treated with a B-spline basis set in analogy with the method presented in ref (99). A cubic grid extending over a 30 Bohr-radius *r*_max_ is employed, with vanishing functions at the boundaries as imposed by the B-splines. Matrix diagonalizations and inversions are performed in the B-spline basis, ensuring smoothness and faithful representation of functions and operators. We checked that 300 B-splines ensure convergence within 1 mRy of the energy values presented throughout. We had to sometimes resort to a smaller basis set of 100 B-splines for more demanding computations, such as those producing the GW+SOSEX potentials. We verified that this does not heavily impact the quality of the results anyway, which remain converged well within a threshold of 10 mRy even with this smaller basis.

Self-energies are calculated using the techniques presented in section 2.4 over a frequency grid to solve eqs 5 and 6. The calculation of LSSE inhomogeneous terms is also presented in section 2.4, but the numerical solution of the LSSE requires more care. The LSSE linear system is rank-deficient,^66^ due to the degree of freedom given by the possibility of incorporating inside the xc potential an arbitrary energy shift. In the spherical problem, this degree of freedom can be saturated by imposing the known theoretical asymptotic behavior
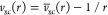
29with *v̅*_xc_ → 0 faster than 1/*r* for *r* → *∞*. In our implementation, we solve for *rv̅*_xc_ with a singular value decomposition (SVD) treatment for the linear system inversion. The SVD allows us to impose the vanishing of *v*_xc_ for *r* → *∞*. Thanks to the B-spline basis set, the unphysical oscillations in the potentials found when solving the problem on a radial grid are avoided.^98,99^ However, we found numerical noise in the matching of *v*_xc_ with the long-range solution −1/*r*. Although the SVD showed some efficacy in removing such noise, we found that it still impacted the quality of the KS HOMOs. We managed to improve them by adopting the following procedure: At each LSSE iteration, we discard *v*_xc_ in the interval [*r*_cut_, *r*_max_], with *r*_cut_ being the radial coordinate at which the noise onset is found, and replace it with −1/*r*; then we shift *v*_xc_ in the leftover interval [0, *r*_cut_] in order to match −1/*r* at *r*_cut_. Unfortunately *r*_cut_ is system-dependent and has to be evaluated for every atom and spin polarization. Nevertheless, this procedure allows us to obtain EXX HOMOs in very good agreement with the HF ones and MP2 and RPA HOMOs in excellent agreement with those in the literature.^98,99^

### 3.2 EXX, MP2, RPA, and GW+SOSEX xc Potentials

The xc potentials for atoms as obtained from the self-consistent solution of eq 8 using the EXX, MP2, and RPA functionals have already been documented and characterized in the literature for a number of atoms, mostly up to Ar.^74,75,98−104^ Here we also consider the solution of eq 8 with the GW+SOSEX self-energy and present calculations for an extended set of spherical atoms, including those from the fourth period (i.e., K, Ca, Mn, Zn, As, and Kr), in which the 3d shell comes into play starting from Mn. Moreover, we extend the RPA calculations previously performed on closed-shell spherical atoms^99^ to spin-polarized spherical atoms having half-filled shells.

**Figure 3:**
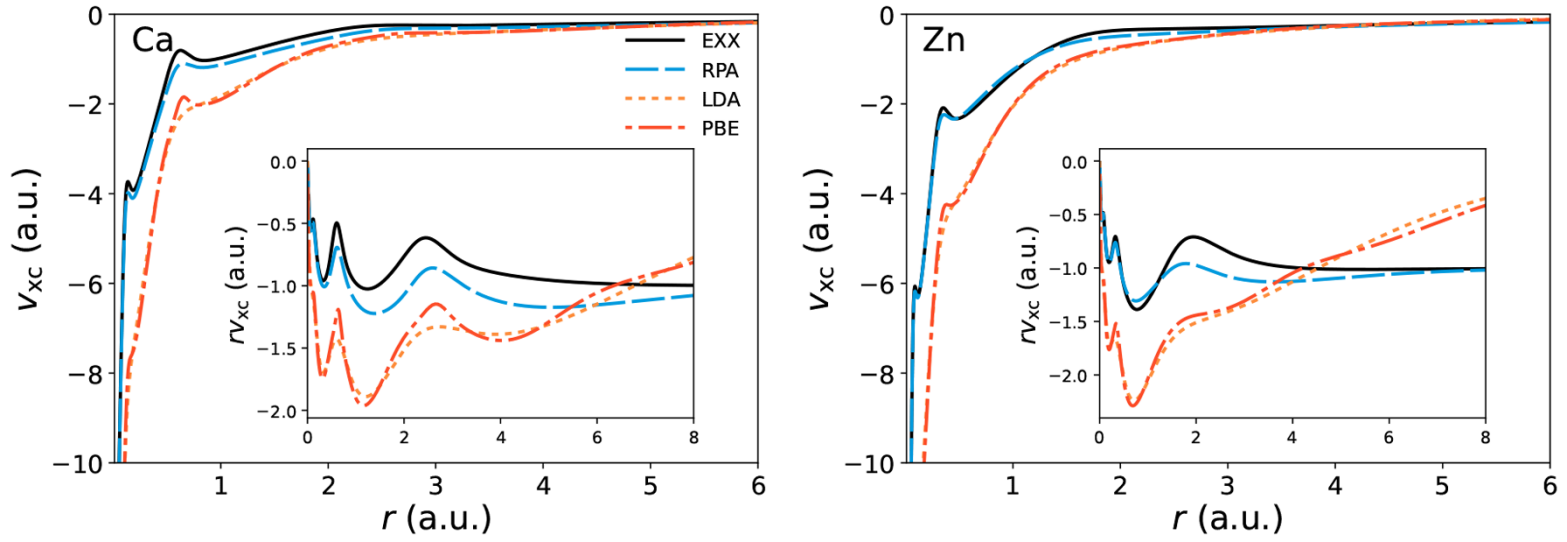
EXX, RPA, LDA, and PBE xc potentials for Ca (left) and Zn (right); (inset) xc potentials multiplied by the radial coordinate.

In Figure 3, we show the potentials for two of the heavier atoms we present in this work, Ca and Zn. The shell structure of the electronic charge in atoms is reflected on the potential through the presence of bumps. These bumps are found between two regions in space where the charge density originates from different orbitals.^79,105,106^ Local functionals used in KS-DFT such as local-density approximation (LDA) cannot describe the charge localization due to the atomic shell structure, displaying smoother behavior in the xc potential. PBE does better in this regard but still yields xc potentials far away from the LSSE solutions, which properly account for exchange. Moreover, neither LDA nor PBE capture the correct 1/*r* decay of the xc potential (insets of Figure 3), which is due to the predominance of exchange in the long-range.^57,58^ Finally, it can also be noticed how filling the shells progressively shortens the range of the potentials, in analogy with the density.

**Figure 4:**
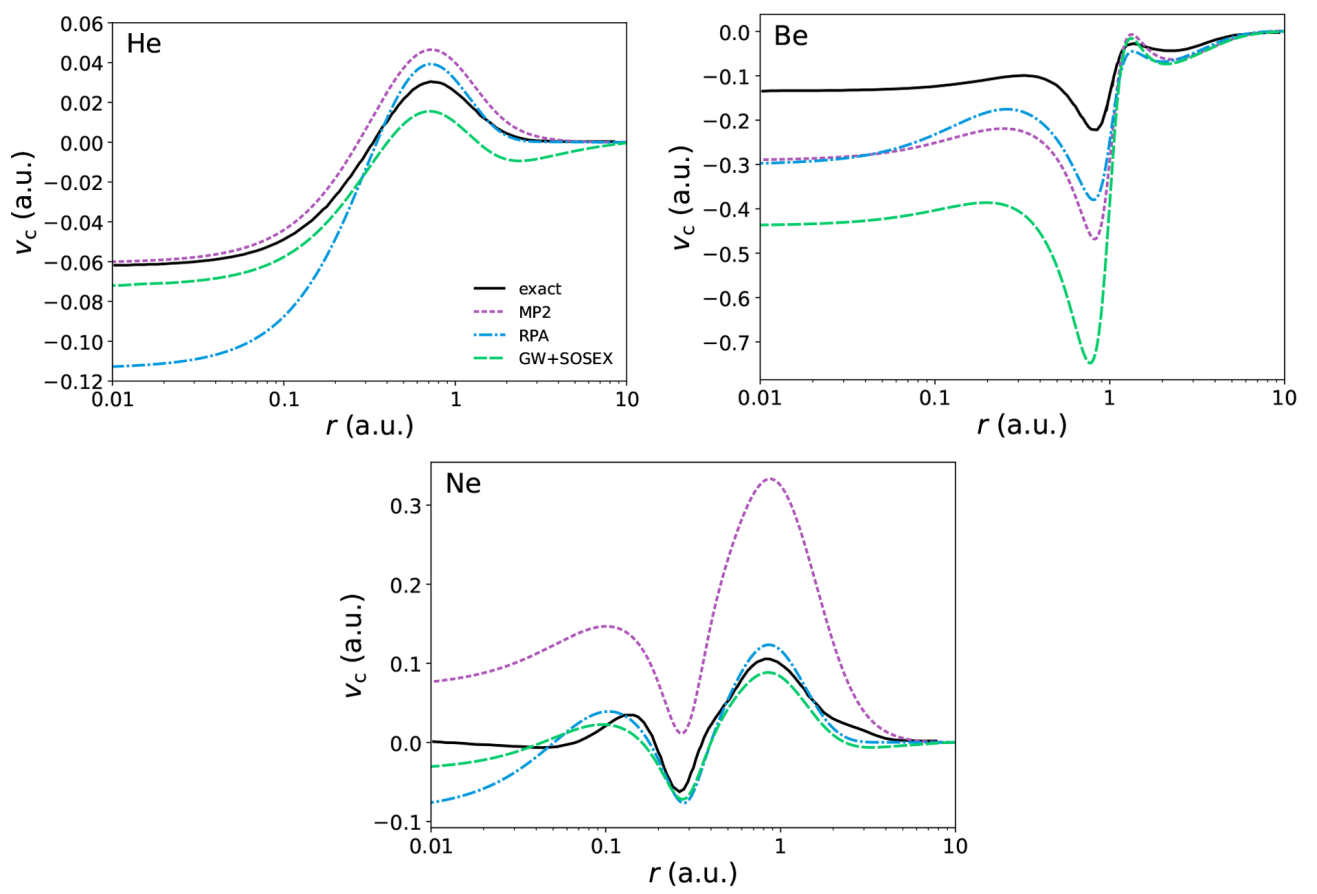
Correlation potentials for helium, beryllium, and neon. The MP2 correlation potential for beryllium is computed on top of EXX orbitals and eigenvalues. Exact correlation potentials are from ref (58).

**Figure 5:**
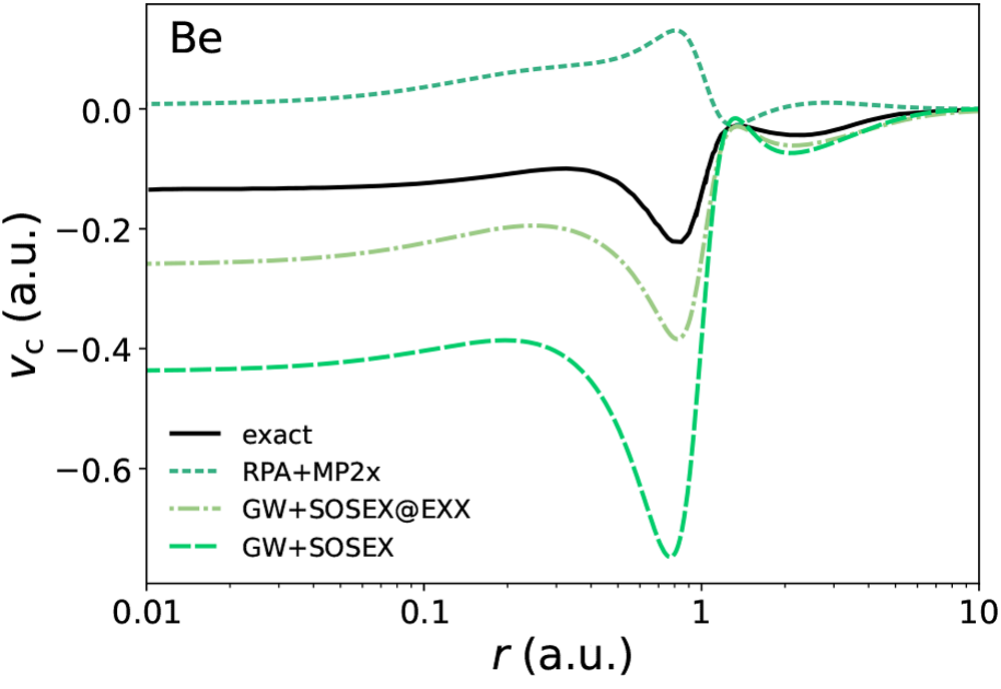
Alternative beyond-RPA correlation potentials for beryllium. Self-consistency has little impact on the GW+SOSEX HOMO: we have ϵ_HOMO_^scGW+SOSEX^ = −0.733 Ry and ϵ_HOMO_^GW+SOSEX@EXX^ = −0.735 Ry. Exact correlation potential is from ref (58).

**Figure 6:**
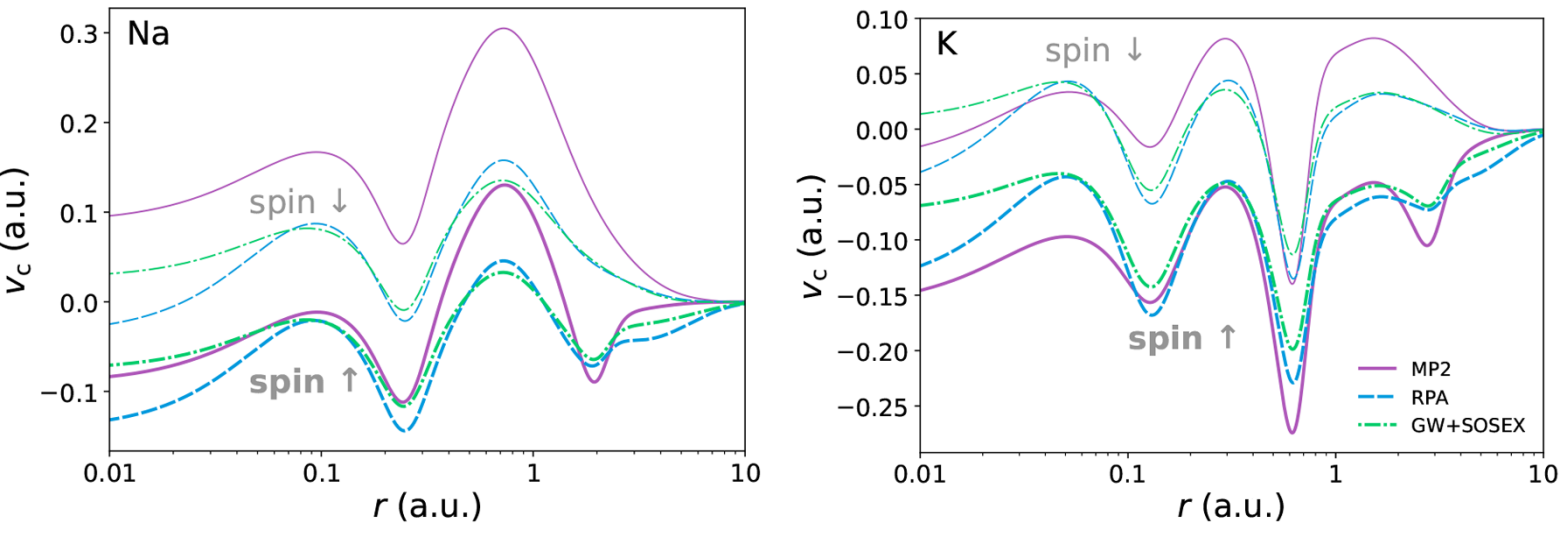
Correlation potentials for sodium and potassium. Thick lines are for the spin majority channel; thin lines are for the spin minority channel.

The inclusion of correlation of any kind generally has the effect of damping the bumps of the xc potential. In order to study correlation with a suitable scale, it is customary to resort to the correlation potential, which is obtained by removing the exchange contribution from the xc potential. Here we adopt the definition of ref (99), relying on the difference between two potentials evaluated at the respective self-consistent densities:
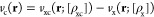
30that is, the potential to be subtracted is the EXX one, *v*_x_ evaluated at its self-consistent density ρ_x_. Some correlation potentials calculated by us are plotted in Figures 4, 5, and 6. For He, Be, and Ne a comparison is made with the exact results obtained by Umrigar and Gonze.^58^ We note that we compare potentials obtained at self-consistency using the respective functionals: this is not the approach adopted by all authors, some preferring to evaluate the potentials with one iteration of the LSSE starting from accurate densities.^98^

In the next section we discuss the IPs as obtained from the KS HOMOs, which can be rationalized observing the behavior of the respective xc potential as displayed in Figure 4. The MP2 functional is known to yield the main features of the exact correlation potential, although often missing the correct scale.^98,103^ For instance, in He the MP2 potential is close to the exact one in the region near the nucleus but poorly matches it in the medium and long range, consistently attaining higher values. As a result, the MP2 HOMO level of He is too shallow and shows little improvement as compared with the EXX one. In contrast, the RPA potential offers a poorer description in the short-range radial region but more closely resembles the exact potential in the middle and long range. This leads to a markedly improved KS eigenvalue, almost exactly matching the one from experiments. In evaluating these results, one should not be fooled by the logarithmic scale commonly adopted for plotting the potentials, which has the effect of extending over a considerable length very small radial regions.

Moving on to Be, we recall that for this atom no self-consistent xc potential can be obtained from the MP2 functional.^98^ The MP2 energy functional is divergent in the presence of a vanishing gap, and the self-consistent procedure leads indeed to the closure of the KS gap. This instability is likely to occur in systems with small KS gaps, as is the case for Be. Ca also presents a small KS gap, and our calculations confirm the presence of the instability also for this atom. As a consequence, the MP2 correlation potentials for Be and Ca are computed by iterating the LSSE solver only once starting from EXX orbitals and eigenvalues. Switching to the RPA functional restores the possibility of having a self-consistent xc potential. It must be noted, however, that the improvement toward the exact potential in Be is modest, as is the improvement of the KS HOMO. Finally, for Ne the MP2 functional produces an especially bad result, whereas the RPA functional performs much better. As we shall see in the next section, the 2B self-energy also performs poorly with Ne. There we discuss why it is likely that it is necessary to fully include screening at least at the RPA level in atoms such as Ne.

We now consider the GW+SOSEX potentials. As a first remark, we observe that the tail of the correlation potential is strikingly off in He, with the zero being approached from below. The issue becomes less relevant in heavier atoms but is still present as one can see in Ne. As a consequence, the GW+SOSEX HOMO levels are generally deeper (with the exception of the group 1 atoms) than the RPA ones, which are already too low as compared with experiment. Even in the short-range region, the GW+SOSEX potential is not always satisfactory: while there is an improvement with respect to RPA in He and Ne, in Be there is a visible worsening. It should be noted, however, that the inclusion of a screened exchange diagram allows for a self-consistent solution in Be, whereas the bare second-order diagram in MP2 did not. If one computes the GW+SOSEX potential for Be with a single iteration of the LSSE on an EXX self-consistent solution, as we did in the MP2 case, a short-range description in line with the ones of MP2 and RPA is recovered (see Figure 5). In this case, self-consistency appears to exacerbate the bad features of the GW+SOSEX potential, as already seen with the MP2 functional.^98^ The KS HOMO is subject to little influence by self-consistency anyway, since it mainly affects the potential in the short-range.

In Figure 5, we also briefly explore the “RPA plus MP2 exchange” (RPA+MP2x) functional in the case of Be, yielding a self-energy consisting of the GW plus 2Bx diagram in the SSE. The resulting xc potential presents features in opposition with those of the exact potential, suggesting that the unscreened 2Bx diagram is not properly balanced by the GW diagram. In He, we even find that the peak in the correlation potential disappears. Overall, all our calculations show that the RPA+MP2x functional performs badly with atoms and should probably not be employed in general. The related GW+2Bx self-energy has also been found to perform poorly in the past.^26^ This suggests that in order to build upon the RPA/GW level of theory some amount of screening should always be included in the higher-order diagrams. Our results show that doing it with the SOSEX diagram restores the features of the exact potential, although retaining a suboptimal asymptotic behavior. The GW+SOSEX diagram can then be viewed more positively and considered as a starting point for more sophisticated self-energies, which could possibly yield better results. For instance, it is an ingredient of the full second-order (in the screened interaction) diagram, which would also restore variationality if employed in the LSSE, it being a Ψ-derivable self-energy.^85,107^ Additionally, a set of diagrams enforcing the positive-definiteness of the spectral function could be obtained starting from the SOSEX diagram, following the recipe provided in ref (40).

Another positive aspect of the GW+SOSEX potential is that it does circumstantially improve on the RPA one. As we discuss in section 3.3, the GW+SOSEX self-energy leads to a better prediction of the HOMO of group 1 atoms (save for H) with either the self-consistent LSSE solution discussed in this section or the one-shot QPE starting from HF (see Figure 8). Therefore, we present in Figure 6 the correlation potentials of two atoms from group 1 (Na and K). We can only speculate on what the improvements of the GW+SOSEX potential are on the RPA one, since no exact potential is currently available for these atoms. Looking at the majority spin-channel potentials, we see that the GW+SOSEX potential is found between the MP2 and the RPA potentials in the medium and long range. From the results of the next section, we can see that for atoms in group 1 the best prediction of the HOMO is given by the MP2 functional. Thus, including a screened exchange diagram, which tends to bring the RPA potential closer to the MP2 one, has the effect of improving the ionization potential, although not making it as accurate as the MP2 one.

### 3.3 Ionization Potentials

**Figure 7:**
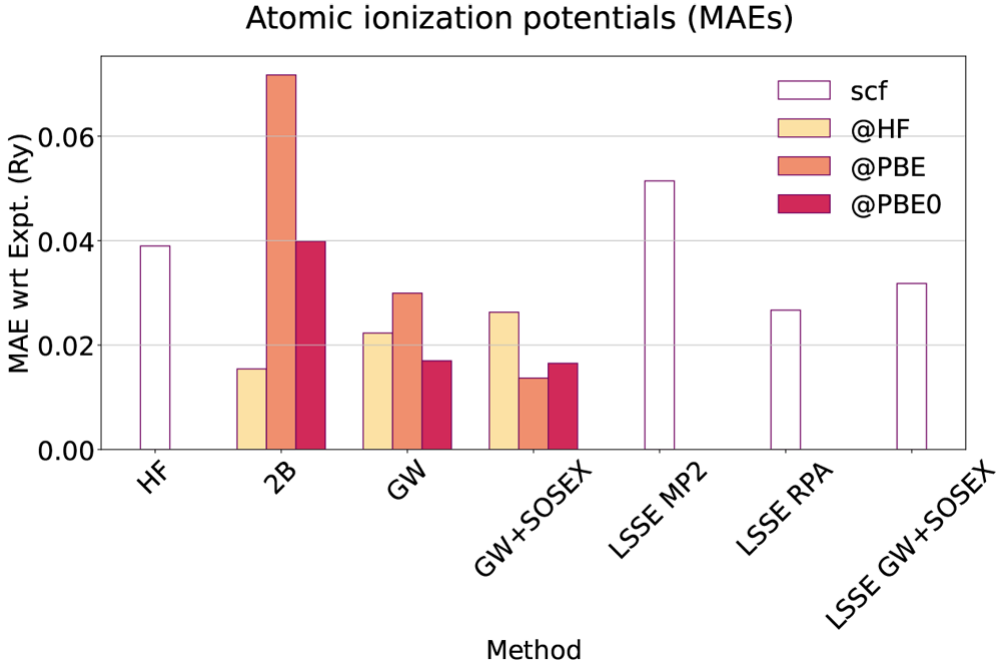
Mean absolute error (MAE) on the ionization potentials for each method and starting point. In white, we indicate the independent-particle self-consistent field (scf) methods.

**Figure 8:**
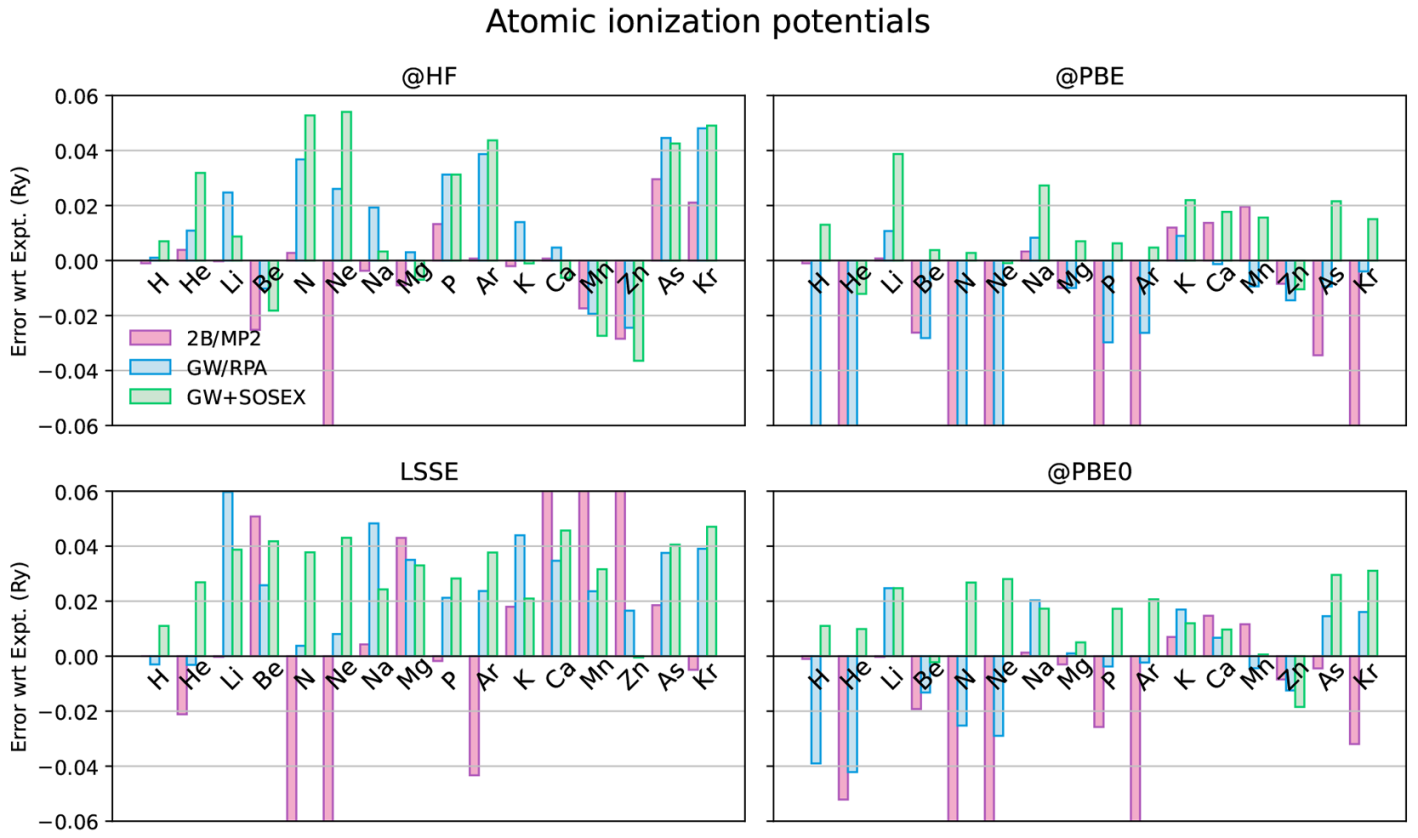
Deviation from experiment of the ionization potentials of the neutral atoms as computed with the selected self-energies and starting points. The LSSE-MP2 IPs of Be and Ca and the LSSE-GW+SOSEX IP of Mn are computed starting from EXX orbitals and eigenvalues. Experimental IPs are from ref (108).

In this section, we address the performance of the different self-energies described in Figure 1. We do this by evaluating the mean absolute error (MAE) with respect to experiment of the IPs computed with each self-energy approximation (Figure 7). These are obtained as the negative of the HOMO energies, that is, IP = −ϵ_HOMO_. In Table 1, we also provide the maximum and minimum errors found on the test set, and in Figure 8, we provide the error contributions resolved on each atom. The LSSE results are considered at the scf level without an iteration of the QPE. In fact, as we discuss at the end of this section, the LSSE does provide a consistent starting point for the QPE, if the same self-energy approximation is used in both steps, so that the QPE produces a mostly negligible QP shift on the HOMO.

**Table 1:** Negative of HOMO Energies (Ry) as Computed with Different Self-Energies and Starting Points: Mean Absolute Error (MAE), Maximum Absolute Error (Max AE), and Minimum Absolute Error (Min AE)

	@HF	@PBE	@PBE0	LSSE
–ϵ_HOMO_^2B^				
MAE	0.015	0.072	0.040	0.051
Max AE	0.088	0.491	0.250	0.273
Min AE	0.000	0.001	0.000	0.000
–ϵ_HOMO_^GW^				
MAE	0.022	0.030	0.017	0.027
Max AE	0.048	0.086	0.042	0.060
Min AE	0.001	0.001	0.001	0.003
–ϵ_HOMO_^GW+SOSEX^				
MAE	0.026	0.014	0.017	0.032
Max AE	0.054	0.039	0.031	0.047
Min AE	0.001	0.001	0.001	0.000

The first notable result is that on average the 2B self-energy performs remarkably well on the studied atoms on top of the HF starting point. However, not considering alkali metals, it falls short of this result if the PBE or PBE0 functionals are used instead, also displaying a strong starting point dependence. We note that the 2B self-energy is generated by one iteration through Hedin’s scheme with the TDHF vertex, and subsequent truncation to second order in the Coulomb interaction. Therefore, employing the HF starting point implies having a consistent functional derivative of the self-energy with respect to the GF in the vertex equation (i.e., a physical polarizability).^36^ This, together with other results from the literature,^23,36,104^ points to a possible relevant role of a consistent inclusion of the vertex corrections, which should be further investigated. This may also explain why, as seen in the previous section, the MP2 functional treated at the LSSE level produces underwhelming results, along with perturbative QPE schemes using starting points different from HF.

We note, however, that with some atoms, such as Ne, the 2B self-energy fails even with the HF starting point. This can be explained with the findings of Bruneval,^52^ who pointed out the sizable difference that including all the infinite GW self-energy diagrams can sometimes make as compared with including just the first single-ring one, that is, the 2Bd diagram in this work. This difference suggests that screening plays an important role also in finite systems, as already emphasized by Shirley and Martin in ref (8). Here, we find it to be especially important in Ne in order to obtain an accurate result. However, this does not imply that the RPA screening on top of a HF solution (RPA@HF), which we are using in GW@HF, is necessarily the best choice. In fact, according to ref (52), the QP shift in N is also sensitive to the full inclusion of the RPA screening beyond the 2Bd diagram via GW. However, 2B performs quite well with N, whereas GW does considerably worse. Including the SOSEX diagram further worsens the result for N, suggesting that adding more diagrams with the RPA@HF screening can do little to improve the description.

The behavior described for N is confirmed for many of the selected atoms: in most cases, GW@HF overbinds the HOMO and GW+SOSEX@HF increases the error. A similar trend has also been recently found using the GW100 test set.^28^ Therefore, the introduction of the SOSEX diagram does not seem justified with the HF starting point, owing to the suboptimal RPA@HF screening. A few notable exceptions are alkali metals, Li, Na, and K: it could be argued that the self-screening of the lone s electron is particularly relevant in these atoms and is thus the main source of error for GW, which can be ameliorated by GW+SOSEX. However, this explanation is not confirmed by the H atom, the archetypical system for self-screening detection: in this case the error of GW@HF is already small to start with and GW+SOSEX@HF does worse. As we shall see, the picture is puzzlingly the opposite when using (generalized) KS-DFT solutions from the PBE and PBE0 functionals as starting points.

Interestingly, the results for GW@HF and GW+SOSEX@HF are generally mirrored in the LSSE framework: GW+SOSEX consistently improves on GW in atoms of group 1 and consistently does worse in almost all the others. This may be explained by the fact that the EXX contribution is dominant in determining the LSSE occupied orbitals and eigenvalues, which end up being similar to the HF ones, especially in the upper valence.^75^ We therefore conclude that the KS spectrum as obtained from the LSSE is not sufficient to provide a better RPA screening than the HF one. The remaining discrepancies between the QPE@HF and the LSSE methods may be explained by the following two major facts: (i) the solution of the LSSE has a different unoccupied spectrum, with the LUMO being a KS bound state, whereas it is an unbound state in QPE@HF; (ii) the LSSE also involves a form of KS self-consistency. As compared with GW@HF and GW+SOSEX@HF, these two differences provide some advantage to the LSSE in noble gases, in atoms of the N family, and in Zn (the LSSE-GW+SOSEX IP of Zn surprisingly matching the experiment) but end up worsening the results in group 1 and 2 atoms.

If solutions using density functionals in the (generalized) KS scheme are used as a starting point, the picture drastically changes. GW@PBE is seen to do worse than GW@HF, now strongly underbinding the HOMO in many lighter atoms. Adding the SOSEX diagram greatly improves the HOMO energies, reducing by half the MAE. The GW+SOSEX self-energy is now seen to perform worst with the atoms with which it performed best using the HF starting point, that is the group 1 atoms; and conversely, GW performs better. Notably, the rationale for the SOSEX diagram as a cure for the self-screening problem of GW seems now justified if H is considered. This confirms early observations that the GW self-screening problem in H can be exacerbated by a starting point that suffers from a self-interaction/delocalization error and is instead small when an accurate starting point is adopted.^50,51^

Going from the PBE to the PBE0 starting point greatly affects the performance of GW, which is now comparable with GW+SOSEX@PBE and scarcely the one of GW+SOSEX. On the one hand, this confirms a reduced starting point dependence of the GW+SOSEX self-energy,^27,28^ also seen in self-consistent schemes.^63^ On the other hand, a fraction of exchange in the starting point might as well be sufficient for obtaining accurate IPs with GW.^14^ Of course one should consider larger test sets for a more reliable assessment: the IPs of ref (27) obtained with the larger G2 test set seem to confirm this picture. The more recent results of ref (28) obtained with the GW100 test set do show a marked improvement with the GW+SOSEX self-energy instead, even with the PBE0 reference. Moreover, other observables such as deeper binding energies may also be considered for a more comprehensive assessment.

We conclude this section by checking the validity of the LSSE starting points as approximate self-consistent solutions. In Table 2, we report the ionization potentials of all atoms as obtained from self-consistent solutions of the LSSE and from the solution of the QPE (eq 6) on top of the LSSE solutions. As expected on the basis of the LSSE construction (preserving the charge density, and therefore the IP, to first order in converting the KS equations into the Dyson equation),^65−67^ it can be seen that 2B@LSSE-MP2 and GW@LSSE-RPA calculations exhibit almost no QP shift in the IPs, the discrepancy between the KS HOMO and the QP HOMO being at most 1 mRy (with the exception of the 2B@LSSE-MP2 calculation for Zn). The discrepancies between the GW+SOSEX HOMOs are more sizable instead but still within 10 mRy (with the exception of Li). The reason why this occurs may be that the GW+SOSEX self-energy is not suitable for the variational argument presented in section 2.2. Moreover, the suboptimal long-range behavior of the GW+SOSEX xc potential may also hint at some underlying numerical issue in the solution of the LSSE. In fact, these potentials are more sensitive to the *r*_cut_ parameter chosen for matching with the long-range solution (see section 3 of the Supporting Information); therefore, a larger error bar is to be expected on the IPs as obtained from the GW+SOSEX HOMO energies than one gets with the MP2 and RPA potentials, however not undermining the assessment presented in this section. One may conservatively estimate this error from the QP shift obtained from the Dyson equation, which, as already mentioned, can be up to the order of 10 mRy.

**Table 2:** IPs (Ry) from the Self-Consistent Solution of the LSSE vs IPs from the One-Shot Solution of the QPE upon the Self-Consistent LSSE^a^

	LSSE			QPE@LSSE			
atoms	MP2	RPA	GW+SOSEX	2B	GW	GW+SOSEX	expt^108^
H	1.000	0.997	1.011	0.999	0.997	1.017	0.999467
He	1.785	1.802	1.833	1.785	1.803	1.841	1.80714
Li	0.396	0.455	0.437	0.396	0.455	0.449	0.39628
Be		0.710	0.733		0.709	0.741	0.68521
N	1.000	1.072	1.106	1.001	1.071	1.113	1.06824
Ne	1.316	1.594	1.629	1.316	1.594	1.639	1.58496
Na	0.381	0.426	0.403	0.381	0.426	0.411	0.37772
Mg	0.604	0.596	0.595	0.603	0.596	0.601	0.56199
P	0.770	0.792	0.799	0.771	0.793	0.807	0.7707575
Ar	1.117	1.183	1.196	1.117	1.183	1.206	1.15831
K	0.333	0.363	0.340	0.333	0.364	0.345	0.31904
Ca		0.483	0.495		0.483	0.503	0.44931
Mn	0.623	0.570		0.623	0.570		0.546390
Zn	0.800	0.707	0.689	0.795	0.707	0.696	0.6904609
As	0.738	0.757	0.759	0.737	0.758	0.766	0.71945
Kr	1.024	1.069	1.075	1.025	1.070	1.082	1.02895

a No self-consistent MP2 xc potentials can be computed for Be and Ca owing to an instability due to the closure of the KS gap in the self-consistency procedure. No self-consistent GW+SOSEX potential for Mn is present either due to numerical issues. Basis set size = 100 B-splines.

In order to check whether the LSSE could be an approximate pathway to self-consistency, it would also be useful to compare the MP2 and RPA IPs with the self-consistent 2B and GW ones, respectively. In Table 3, we compare our IPs from the solution of the LSSE with those from the self-consistent calculations of refs (9 and 46) (see also section 1 of the Supporting Information). We find a generally poor agreement of the 2B/MP2 IPs and a partial agreement of the GW/RPA ones, that is, only for He and Ne do we have similar GW/RPA IPs. While some aspects of our numerical treatment may differ from those of ref (46), this does not seem sufficient to explain so-large a discrepancy between the 2B and MP2 results. Therefore, it can be argued that the LSSE does not provide a good approximation to the self-consistent Dyson equation when the 2B self-energy is employed. The agreement between the two methods appears to be sometimes better with the GW self-energy but still system-dependent. Further precise self-consistent calculations are needed to assess when and whether the two methods yield similar results. Other observable quantities should also be targeted in the LSSE approximate self-consistency, such as the HOMO–LUMO gap upon inclusion of the derivative discontinuity correction.^70^

**Table 3:** Self-Consistent LSSE RPA and MP2 IPs (Ry) of Selected Atoms Compared to Self-Consistent GW and 2B Ionization Potentials from the Literature

atoms	LSSE-RPA (B-spline)	sc-GW^9^ (Slater orb.)	LSSE-MP2 (B-spline)	sc-2B^46^ (Slater orb.)	expt^108^
He	1.804	1.805	1.786	1.811	1.80714
Be	0.711	0.636		0.660	0.68521
Ne	1.593	1.600	1.312	1.497	1.58496
Mg	0.597	0.535	0.605	0.553	0.56199

## 4 Conclusions and Perspectives

In this work, we have presented a benchmark of three self-energies derived within MBPT, that is, 2B, GW, and GW+SOSEX. We implemented the resolution of the many-body QPE and of the self-consistent LSSE with these self-energies for a set of spherical atoms, aided by an easily converged B-spline basis set. We chose to also consider the solution of the LSSE for two reasons: to possibly have a consistent starting point for the QPE and, as a further benchmark, to compare the xc potentials we get from it with the exact ones available in the literature.

The EXX, MP2, and RPA xc potentials computed by solving the LSSE reproduce the ones present in the literature. We extended these calculations to atoms belonging to the fourth period and to spin-polarized cases, the latter kind not yet considered for the RPA potential. Furthermore, we explored the GW+SOSEX self-energy in the LSSE framework: we find that it brings the correlation potentials closer to the exact ones in the short-range for He and Ne but not for Be; in the long-range, the description is even worse, with these potentials reaching zero from below. This is especially noticeable in lighter atoms, whose HOMO is affected worst by this bad feature. In contrast, the GW+SOSEX potential seems to improve on GW in group 1 atoms by bringing the description closer to the MP2 one.

The bottom line of this part of the work is that, as is known in MBPT, also in the LSSE framework “more diagrams” does not necessarily mean “better results”: the GW+2Bx/RPA+MP2x calculations we briefly presented are explanatory in this regard. At least we find the GW+SOSEX potentials to restore the correct main features of the exact potentials by building upon the GW+2Bx/RPA+MP2x level of theory. Therefore, we believe that improving on the GW+SOSEX level of theory with a sensibly chosen criterion could fix the issue of the long-range behavior. Among these criteria, we include variationality (e.g., considering the self-energy diagram of second order in the screened interaction) or positive definiteness of the spectral function (following ref (40)).

Concerning the IPs, we find the solutions of the LSSE to provide a consistent starting point for the QPE, yielding small to vanishing QP shifts in the HOMO and therefore hinting at conservation of the starting density to a large degree.^67^ However, the comparison with the existing literature, though limited, suggests that this does not necessarily mean that the IPs we obtain from the self-consistent LSSE are a good approximation to the ones obtained from the self-consistent Dyson equation. We also find that the LSSE generally performs poorly as compared with the resolution of the QPE starting from independent-particle solutions given by either the HF method or semilocal/hybrid functionals in (generalized) KS-DFT. In this regard, we get the best performance from GW+SOSEX@PBE/PBE0, 2B@HF, and GW@PBE0. This confirms the common knowledge that hybrid functionals provide good starting points for GW applied to finite systems,^11^ even in the atomic limit.

The justification of the SOSEX diagram as a cure for the self-screening error of GW seems to be correct only with the PBE and PBE0 starting points. On the contrary, adding the SOSEX diagram to GW with the HF starting point appears to be inaccurate, likely due to the poor description given by the RPA screening computed on top of a HF solution. These performances are in line with those found with larger test sets involving small molecules.^27,28^ However, we must point out that, as in ref (27), we find that the GW+SOSEX self-energy does not bring a marked improvement to the IPs, on average, as compared to GW, when the PBE0 reference is used; analogous results have been recently found by Bruneval et al. in ref (14) by using another hybrid reference, leading them to claim that GW is still the best choice within MBPT for computing IPs of molecules. Almost as recently, and by using the same test set, the work by Wang et al. presented in ref (28) has instead shown an improvement on GW with the GW+SOSEX self-energy even starting from the PBE0 reference. As we already stated in the introduction, the arbitrariness of the starting point adds a layer of complexity to the assessment of a self-energy-based method, and the possibility of tuning a parameter in hybrid functionals seems to allow GW to be most often up to the task,^14,109,110^ perhaps at the expense of conceptual clarity.

Finally, we are positively impressed by the good performance of the 2B self-energy. This motivates us to build upon this level of theory by including all the self-energy diagrams generated by the TDHF vertex, which has recently been shown to be a promising route to electronic excitations in molecules.^36,37^

## Figures and Tables

**Figure 9:**
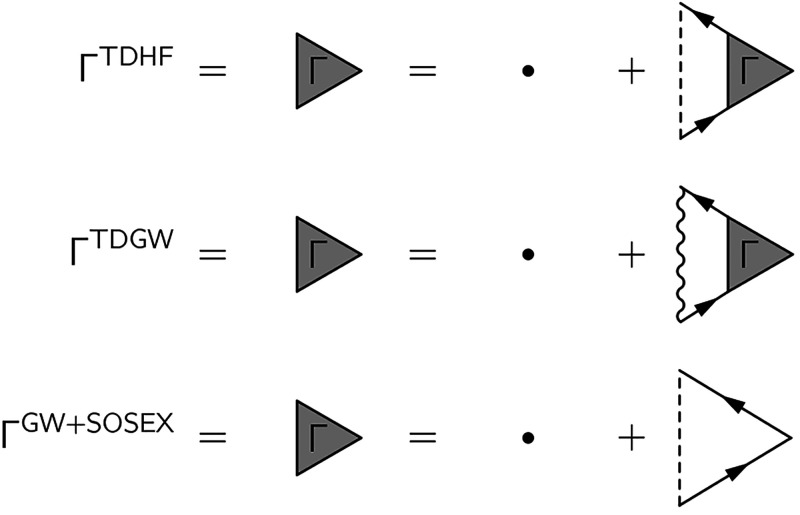
Vertex equations in the TDHF, TDGW, and GW+SOSEX approximations.

**Figure 10:**
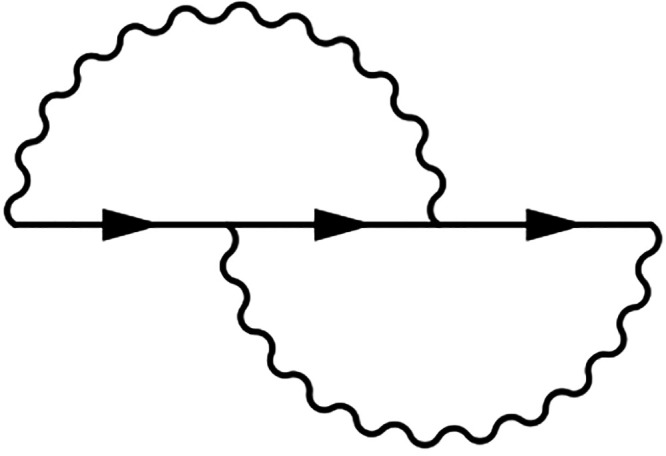
Self-energy diagram of order 2 in the screened interaction *W*.

## Appendix A: Self-Energies from Vertex Corrections

Iterating through Hedin’s scheme with a nontrivial vertex function would in principle be an ideal and systematic way to devise beyond-GW approximations. However, doing so can quickly lead to very complex methods. This procedure allows for the inclusion of self-energy diagrams to infinite order. Nevertheless, since the vertex function includes the derivative of the self-energy, Σ, with respect to the Green’s function *G*, new diagrams are added at each iteration, making a rigorous implementation of Hedin’s scheme numerically unfeasible. In order to overcome this complexity, approximations and truncations in the iteration process must be made. In the GW approximation, vertex corrections are neglected altogether, making the vertex function trivial and allowing for a self-consistent solution independent of the starting point.

The simplest vertex correction is the time-dependent Hartree–Fock (TDHF) scheme, obtained by plugging the Fock self-energy into the vertex equation (see Figure 9, top). This vertex has been studied in a perturbative fashion, that is, with a single or a few iterations through Hedin’s scheme, starting from a HF reference.^8,36,37^ By plugging the GW self-energy into the vertex function, the time-dependent GW (TDGW) method is obtained instead (see Figure 9, middle). For simplicity, the dependence of the screened interaction on the Green’s function is generally ignored in the differentiation contained in the vertex function. By doing so, a TDGW vertex is obtained, leading to the same skeleton self-energy diagrams generated by the TDHF vertex, but framed in terms of the screened interaction.

A way to further simplify the scheme is to truncate the infinite summation of self-energy diagrams generated by a vertex function, retaining only lower orders. This allows one to avoid the often cumbersome solution of the vertex equation and simply compute a number of selected self-energy diagrams. For instance, with truncation to second order in the Coulomb interaction, the summation generated by the TDHF vertex yields the second Born (2B) self-energy. With truncation to second order in the screened Coulomb interaction, the summation generated by the TDGW vertex yields the GW self-energy plus a second-order exchange contribution, the latter corresponding to the 2B exchange (2Bx) diagram expressed in terms of the dressed interaction *W* (see Figure 10). When this diagram is implemented, the two screened interactions have most often been considered statically^34^ or with a plasmon pole approximation;^33,38^ only recently has their frequency dependence been fully taken into account.^23,28^ Moreover, a treatment for this diagram enforcing positive definite spectral functions has also been proposed.^40−42^

A simpler second-order approximation, devoid of double frequency integrals, can be devised by retaining only the zeroth and first-order terms in the TDHF vertex (see Figure 9, bottom) and then substituting them into the self-energy. This produces a second-order diagram with one screened Coulomb interaction, leaving the other at the bare level. This diagram is termed the second-order screened exchange (SOSEX) diagram and the self-energy approximation is referred to as GW+SOSEX. This has been studied so far for molecules^27,28,62^ and model systems.^63^

## Supporting Information Available

Comparison of results with existing literature, convergence of the xc potentials, and perturbative versus self-consistent solution of the LSSE (PDF)
