# Supplementary material for: Numerically Precise Benchmark of Many-Body Self-Energies on Spherical Atoms
Source: J Chem Theory Comput. 2022 May 13;18(6):3703–17. doi: 10.1021/acs.jctc.2c00048 (PMC9202310; doi:10.1021/acs.jctc.2c00048)
Supplement: Supplementary file 1 — ct2c00048_si_001.pdf [file ct2c00048_si_001.pdf]

Supporting Information to Numerically precise benchmark of many-body self-energies on spherical atoms

S. Vacondio,[*,†,‡] D. Varsano,[‡] A. Ruini,[†,‡] and A. Ferretti[‡]

†*Dipartimento di Scienze Fisiche, Informatiche e Matematiche, Università di Modena e Reggio Emilia, Via G. Campi 213/a, Modena, Italy*

‡*Centro S3, CNR–Istituto Nanoscienze, 41125 Modena, Italy*

E-mail: simone.vacondio@unimore.it

1 Comparison with the existing literature

Since our results are easily converged thanks to the B-spline basis set we employ, it is preferable to verify them against data affected by an error as small as possible coming from basis set incompleteness. This seems to be the case for the data in Refs. 1–3, where GW and 2B calculations are presented. In Ref. 1 a basis set consisting of 30-40 Slater orbitals is used; the basis set of Ref. 2 is presumably analogous. In Ref. 3 a basis set consisting of numeric atom-centered orbitals is employed, enriched with an auxiliary basis instrumental in the resolution of the identity technique; the calculations are performed with the 'tier 4 + a5Z-d' set, except the ones for noble gases, with which the 'tier 4 + aug-cc-pV5Z' set is used instead.

Table 1 reports the GW@HF and the 2B@HF IPs of our work as compared with these references; Table 2 in the main text does the same with the self-consistent results. Overall,

Table 1: GW and 2B ionization potentials (Ry) of selected atoms computed using different codes wih the HF starting point.
†'tier 2 + aug-cc-pV5Z' for noble gases.

**Atoms**	GW@**HF**			2B@**HF**			**Exp.**[4]
	This work (B-spline)	FHI-AIMS[3] (tier 4 + a5Z)†	Ref. 2 (Slater orb.'s)	This work (B-spline)	FHI-AIMS (tier 4 + a5Z)†	Ref. 1 (Slater orb.'s)	
H	1.001	1.000		0.999	1.000		1.000
He	1.818	1.814	1.819	1.811	1.807	1.8118	1.80714
Li	0.421	0.417		0.396	0.395		0.39628
Be	0.675	0.673	0.675	0.660	0.660	0.6550	0.68521
N	1.105	1.091		1.071	1.101		1.06824
Ne	1.611	1.599	1.610	1.497	1.486	1.4726	1.58496
Na	0.397	0.395		0.374	0.374		0.37772
Mg	0.565	0.556	0.565	0.553	0.547	0.5210	0.56199
P	0.802	0.788		0.784	0.792		0.7707575
Ar	1.197	1.182		1.159	1.147		1.15831

Table 2: GW and GW+SOSEX ionization potentials (Ry) of selected atoms computed using different codes wih the PBE starting point.

**Atoms**	**GW@PBE**		**GW+SOSEX@PBE**		**Exp.**[4]
	This work (B-spline)	FHI-AIMS[5] (tier 4 + a5Z)	This work (B-spline)	FHI-AIMS (tier 4 + a5Z)	
H	0.917	0.920	1.015	1.014	1.000
He	1.722	1.734	1.795	1.791	1.80714
Li	0.405	0.417	0.435	0.434	0.39628
Be	0.657	0.664	0.689	0.697	0.68521
N	0.997	0.997	1.071	1.067	1.06824
Ne	1.514	1.510	1.584	1.560	1.58496
Na	0.383	0.401	0.405	0.400	0.37772
Mg	0.552	0.567	0.569	0.579	0.56199
P	0.743	0.743	0.777	0.772	0.7707575
Ar	1.132	1.118	1.163	1.145	1.15831

the GW@HF results compare well with both the results obtained with the 'tier 4 +a5Z' basis and those obtained with the Slater orbital basis. In the latter case the agreement is notably up to 1 mRy. The situation is different for the 2B@HF results: a good agreement is found again with those from the 'tier 4 + a5Z' basis, but it is worse than before with those from the Slater orbital basis. As the atomic number increases, the agreement worsens noticeably with the latter basis. While the GW results by the same authors compare extremely well

with ours, the 2B ones do not, and we have not found an explanation to this.

In Table 2 the comparison is repeated with the ’tier 4 + a5Z’ results, this time using GW@PBE and GW+SOSEX@PBE IPs from Ref. 5. As with the HF starting point, a good agreement is obtained once more, this time also considering the GW+SOSEX self-energy.

2 Convergence of the xc potentials

Table 3: Negative of RPA HOMO energies (Ry), 100 B-splines vs 300 B-splines.

**Atoms**	$-\epsilon^{\mathbf{RPA}}_{\mathbf{HOMO}}$	
	100 B-splines	300 B-splines
He	1.8023371	1.8029515
Li	0.4550431	0.4557226
Be	0.7100912	0.7102626
N	1.0716163	1.0709971
Ne	1.5939843	1.5927099

Table 4: Negative of GW+SOSEX HOMO energies (Ry) at various basis set sizes.

**Atoms**			$-\epsilon^{\mathbf{GW+SOSEX}}_{\mathbf{HOMO}}$		
	100 B-splines	150 B-splines	200 B-splines	250 B-splines	300 B-splines
He	1.8330167	1.8334037	1.833606	1.8336913	1.8334226
Li	0.4370556	0.4377825	0.437719	0.4377408	
Be	0.7333055	0.7330347	0.732950		
N	1.1059214	1.1053998	1.105304		
Ne	1.6285281	1.6276265	1.627299		

Table 5: r_{cut}-dependence (Bohr) of the negative of the EXX HOMO energies (Ry). $r_{\text{cut max}} = 15.0, 20.0, 15.0$ Bohr for He, Be, and Ne respectively in EXX calculations.

**Atoms**		$-\epsilon^{\mathbf{EXX}}_{\mathbf{HOMO}}$		
	$r_{\text{cut max}} - 3$	$r_{\text{cut max}} - 2$	$r_{\text{cut max}} - 1$	$r_{\text{cut max}}$
He	1.8351883	1.8351883	1.8351883	1.8351883
Be	0.6182464	0.6182463	0.6182459	0.6182475
Ne	1.7028474	1.7029690	1.7030555	1.7031219

Table 6: r_{cut}-dependence (Bohr) of the negative of the RPA HOMO energies (Ry). $r_{\text{cut max}} = 10.0, 17.0, 9.0$ Bohr for He, Be, and Ne respectively in RPA calculations. Basis set size = 100 B-splines.

**Atoms**		$-\epsilon^{\mathbf{RPA}}_{\mathbf{HOMO}}$		
	$r_{\text{cut max}} - 3$	$r_{\text{cut max}} - 2$	$r_{\text{cut max}} - 1$	$r_{\text{cut max}}$
He	1.8023621	1.8023216	1.8023224	1.8023378
Be	0.7102538	0.7101669	0.7101136	0.7100912
Ne	1.5942515	1.5940522	1.5939822	1.593984

Table 7: r_{cut}-dependence (Bohr) of the negative of the GW+SOSEX HOMO energies (Ry). $r_{\text{cut max}}$ = 10.0, 20.0, 17.0, 15.0, 9.0, 12.0 Bohr for He, Li, Be, N, Ne and Ar respectively in GW+SOSEX calculations (spin majority channel). Basis set size = 100 B-splines.

**Atoms**	$-\epsilon_{\textbf{HOMO}}^{\textbf{GW+SOSEX}}$			
	$r_{\text{cut max}} - 3$	$r_{\text{cut max}} - 2$	$r_{\text{cut max}} - 1$	$r_{\text{cut max}}$
He	1.8292974	1.8308499	1.8319844	1.8330167
Li	0.4386851	0.4381058	0.4375153	0.4370556
Be	0.7309687	0.7318101	0.7326009	0.7333055
N	1.1040543	1.1046918	1.1052843	1.1059214
Ne	1.6231987	1.6253215	1.6271054	1.6285281
Ar	1.1934227	1.1945955	1.1953779	1.1961765

3 Perturbative vs self-consistent solution of the LSSE

Table 8: Negative of HOMO energies (Ry): perturbative LSSE on EXX solutions (@EXX) vs self-consistent LSSE (sc). Basis set size = 100 B-splines

**Atoms**	$-\epsilon_{\mathbf{HOMO}}$					
	MP2		RPA		GW+SOSEX	
	@EXX	sc	@EXX	sc	@EXX	sc
He	1.786	1.785	1.805	1.802	1.833	1.833
Li	0.396	0.396	0.452	0.455	0.435	0.437
Be	0.735		0.720	0.710	0.735	0.733
N	1.009	1.000	1.083	1.072	1.111	1.106
Ne	1.349	1.316	1.581	1.594	1.616	1.629
Na	0.378	0.381	0.422	0.426	0.401	0.403
Mg	0.607	0.604	0.601	0.596	0.600	0.595

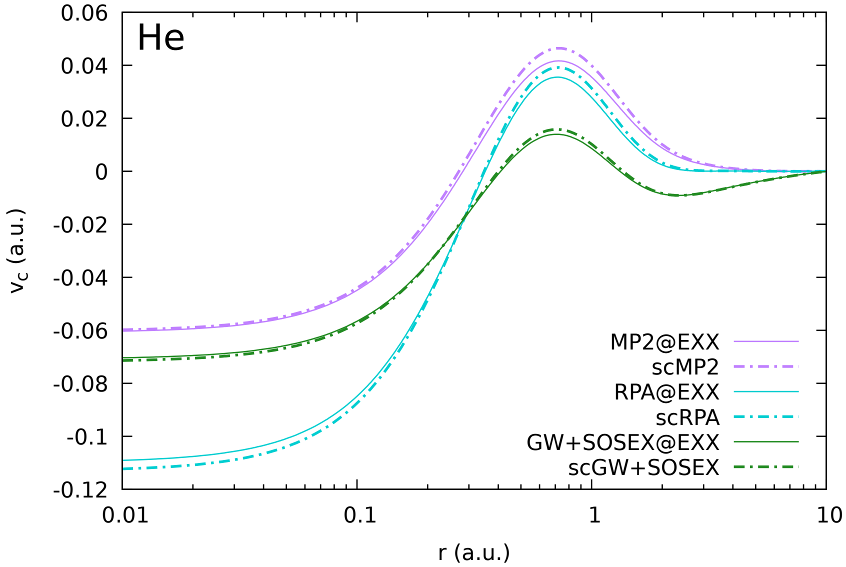

Figure 1: MP2, RPA and GW+SOSEX xc potentials of He: perturbative LSSE on EXX solutions (@EXX) vs self-consistent LSSE (sc).

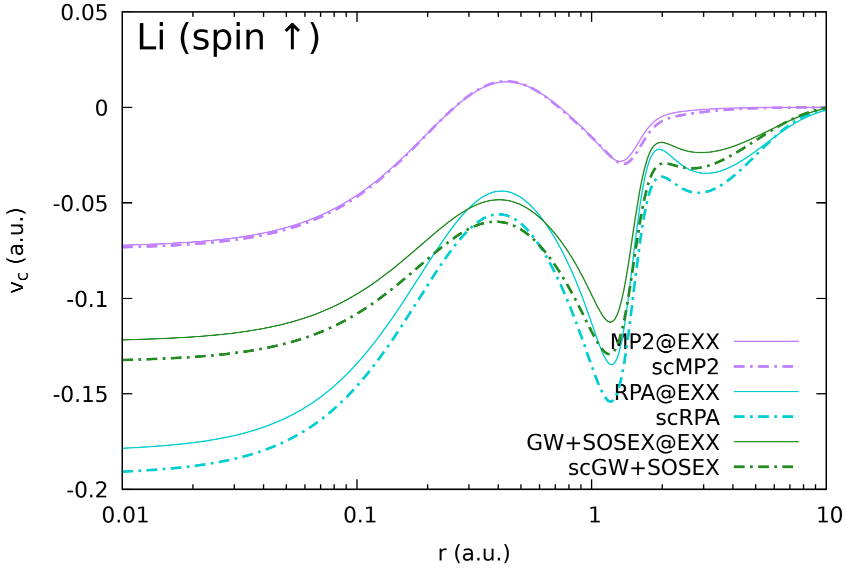

Figure 2: MP2, RPA and GW+SOSEX xc potentials of Li: perturbative LSSE on EXX solutions (@EXX) vs self-consistent LSSE (sc).

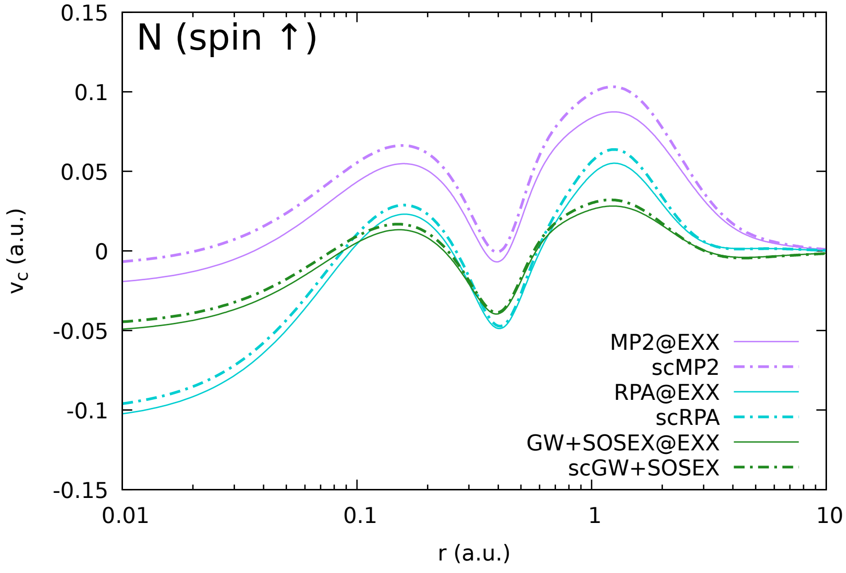

Figure 3: MP2, RPA and GW+SOSEX xc potentials of N: perturbative LSSE on EXX solutions (@EXX) vs self-consistent LSSE (sc).

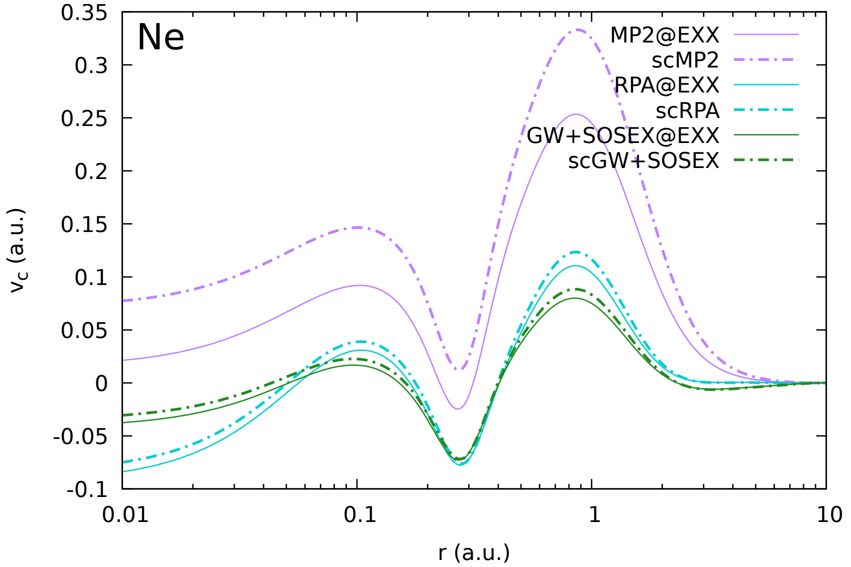

Figure 4: MP2, RPA and GW+SOSEX xc potentials of Ne: perturbative LSSE on EXX solutions (@EXX) vs self-consistent LSSE (sc).

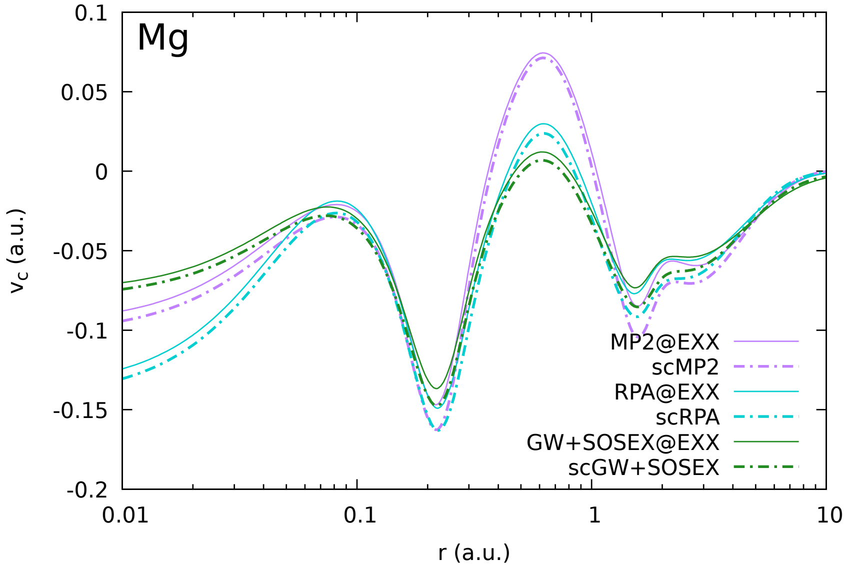

Figure 5: MP2, RPA and GW+SOSEX xc potentials of Mg: perturbative LSSE on EXX solutions (@EXX) vs self-consistent LSSE (sc).

References

(1) Dahlen, N. E.; van Leeuwen, R. Self-consistent solution of the Dyson equation for atoms and molecules within a conserving approximation. *J. Chem. Phys.* **2005**, *122*, 164102, Publisher: American Institute of Physics.

(2) Stan, A.; Dahlen, N. E.; Leeuwen, R. v. Fully self-consistent GW calculations for atoms and molecules. *EPL* **2006**, *76*, 298, Publisher: IOP Publishing.

(3) Ren, X.; Rinke, P.; Blum, V.; Wieferink, J.; Tkatchenko, A.; Sanfilippo, A.; Reuter, K.; Scheffler, M. Resolution-of-identity approach to Hartree–Fock, hybrid density functionals, RPA, MP2 and GW with numeric atom-centered orbital basis functions. *New J. Phys.* **2012**, *14*, 053020, Publisher: IOP Publishing.

(4) Rumble, J. R., Ed. *CRC Handbook of Chemistry and Physics*; CRC Press, 2010.

(5) Ren, X.; Marom, N.; Caruso, F.; Scheffler, M.; Rinke, P. Beyond the GW approximation: A second-order screened exchange correction. *Phys. Rev. B* **2015**, *92*, 081104.
